# Advancing Roles and Therapeutic Potentials of Pyroptosis in Host Immune Defenses against Tuberculosis

**DOI:** 10.3390/biom14101255

**Published:** 2024-10-04

**Authors:** Jiayi Yang, Yuhe Ma, Jiaqi Yu, Yilin Liu, Jiaojiao Xia, Xinen Kong, Xiaoying Jin, Jiaxiang Li, Siqi Lin, Yongdui Ruan, Fen Yang, Jiang Pi

**Affiliations:** 1 Acupuncture and Moxibustion Department, The First Dongguan Affiliated Hospital, School of Medical Technology, Guangdong Medical University, Dongguan 523808, China; jiayi1104yang@163.com (J.Y.); 15625857518@163.com (Y.M.); 13822477577@163.com (J.Y.); liyil6101015@163.com (Y.L.); 19570038268@163.com (X.K.); jxy2799.com@outlook.com (X.J.); 15716338367@163.com (J.L.); linsiqi1125@163.com (S.L.); 13829202566@139.com (Y.R.);; 2 Department of Biochemistry and Molecular Biology, School of Basic Medical Sciences, Kunming Medical University, Kunming 650500, China; 20230052@kmmu.edu.cn

**Keywords:** pyroptosis, tuberculosis, therapeutic, inflammation

## Abstract

Tuberculosis (TB), an infectious disease caused by Mycobacterium tuberculosis (Mtb) infection, remains a deadly global public health burden. The use of recommended drug combinations in clinic has seen an increasing prevalence of drug-resistant TB, adding to the impediments to global control of TB. Therefore, control of TB and drug-resistant TB has become one of the most pressing issues in global public health, which urges the exploration of potential therapeutic targets in TB and drug-resistant TB. Pyroptosis, a form of programmed cell death characterized by cell swelling and rupture, release of cellular contents and inflammatory responses, has been found to promote pathogen clearance and adopt crucial roles in the control of bacterial infections. It has been demonstrated that Mtb can cause host cell pyroptosis, and these host cells, which are infected by Mtb, can kill Mtb accompanied by pyroptosis, while, at the same time, pyroptosis can also release intracellular Mtb, which may potentially worsen the infection by exacerbating the inflammation. Here, we describe the main pathways of pyroptosis during Mtb infection and summarize the identified effectors of Mtb that regulate pyroptosis to achieve immune evasion. Moreover, we also discuss the potentials of pyroptosis to serve as an anti-TB therapeutic target, with the aim of providing new ideas for the development of TB treatments.

## 1. Introduction

Tuberculosis (TB), an infectious disease caused by Mycobacterium tuberculosis (Mtb) infection transmitted via the inhalation of bacteria-laden droplets in the air by those who are infected with TB, remains a deadly global public health burden [1]. TB is one of the leading causes of death worldwide from a single infectious agent, ranking above HIV/AIDs until the coronavirus (COVID-19) pandemic, and is also the leading cause of death among people living with HIV [2]. After Mtb infection, most people (about 20 billion worldwide) develop an asymptomatic latent TB (LTBI) infection, but 5–10% of them will develop active TB (ATB) diseases [2]. Chemotherapy is the primary clinical treatment for TB, but approximately 20% of TB isolates globally are resistant to at least one major drug (e.g., rifampicin, isoniazid). In addition, multidrug-resistant (MDR) and extensively drug-resistant (XDR) TB have emerged, which may further evolve to drug-incurable or programmatically incurable TB in some TB-endemic countries [3]. Thus, the control of TB and drug-resistant TB has become one of the most urgent issues for global public health.

TB typically infects the lungs (pulmonary TB) but can occur as a disseminated disease of other organs and tissues. When Mtb enters the lungs via inhalation, reaching alveolar space, alveolar macrophages (AMs) will rapidly engulf Mtb as the key replication niche for Mtb [4,5]. As an important part of the innate immune system, the macrophages will try their best to kill the intracellular Mtb through different kinds of host immune responses. In response to that, Mtb takes a myriad of strategies to evade the host’s immune responses. Mtb can avoid and/or tolerate phagolysosome formation by inhibiting the maturation of phagosomes and inhibiting the acidification of phagosomes, thus achieving survival inside phagocytes [6]; Mtb can manipulate the death modes of macrophages, such as by inhibiting the apoptosis and autophagy of the infected macrophages while promoting the necrosis and ferroptosis of the infected macrophages. Mtb also can survive in conditions limiting growth, such as hypoxia and lack of nutrients, by activating certain genes [6,7]; Mtb can directly or indirectly prevents the activation of immune cells and bactericidal action by surface or secreted substances [8,9,10,11,12], or reduce the immune effect by preventing or delaying antigen presentation [13,14]. These responses thus lead macrophages to fail to inhibit or destroy the intracellular Mtb, and the bacteria will multiply within the intracellular spaces or even be released, allowing for the infection of other macrophages [15]. The release of inflammatory cytokines by pyroptosis recruits monocytes, epithelioid cells, etc., to the site of infection and contributes to granuloma formation with the aim of converting bacterial phagosomes into acidified antimicrobial compartments for the resistance of Mtb [16], and maybe contributes to granulomatous inflammation or even granuloma rupture [17].

Pyroptosis was redefined as the programmed cell death of plasma membrane pores, formed by gasdermin protein family members, by the Nomenclature Committee on Cell Death (NCCD) in 2018 [18]. As a different form of programmed cell death compared with apoptosis, pyroptosis can trigger inflammatory responses that are not triggered in apoptosis. These inflammatory responses are not limited to granulomatous inflammation, but the IL-1β and IL-18 produced during pyroptosis attract neutrophils, macrophages and other immune cells to the site of infection, resulting in rapidly occurring local acute inflammation or a systemic or chronic inflammatory response via the bloodstream. Pyroptosis is regulated by a group of crucial inflammatory caspases [19], which can cleave a gasdermin protein to release its N-terminal domain to form cell membrane pore-mediated gasdermin proteins and mature IL-1β and IL-18 [20,21,22], and eventually lead to cell membrane rupture and the release of inflammatory contents, including IL-1β and IL-18 [23,24]. Currently, a lot of studies have shown that pyroptosis is widely involved in the development of tumors (e.g., breast cancer) [25,26,27,28,29], cardiovascular diseases (e.g., atherosclerosis) [23,30,31], neurological disorders (e.g., Alzheimer’s disease) [29,32], metabolic disorders (e.g., diabetes), infectious diseases (e.g., TB), etc. [29,33,34], revealing the potential to develop new therapeutic avenues by targeting pyroptosis.

In the innate immune system, AMs serve as the earliest replicative ecological niche for Mtb, and upon infection by Mtb, ESAT-6 secretion system 1 (ESX-1) induces inflammasome activation and, subsequently, the formation of caspase-1 can cleave gasdermin-D (GSDMD) pores and the release of IL-1β. Mtb-infected AMs that do not undergo inflammasome activation are recruited to the lung interstitium by IL-1 released from airway-resident AMs, where cell aggregation initiates the inflammatory response and granulomas are formed, limiting Mtb dissemination. However, Mtb has evolved to be highly adapted to persist within host cells or to achieve immune escape, releasing from host cells by a variety of mechanisms to extend the range of infection. Simultaneously, non-selective or uncontrolled pyroptosis exacerbates the inflammatory response, leading to severe tissue damage and organ dysfunction, which can worsen the outcome of TB infection. Consequently, pyroptosis can thus be regarded as a “double-edged sword” in Mtb infection and TB progression.

Here, we summarize the mechanisms of pyroptosis, as well as the roles and regulatory mechanisms of pyroptosis in Mtb infection and TB progression. By focusing on the four main pathways of pyroptosis and how Mtb promotes the pyroptosis pathway or implements immune escape, this review also provides a brief overview of the current application of pyroptosis for disease treatment, suggests a number of ideas that may be considered to serve pyroptosis as a target for TB treatment, in particular the need to find a balance between promoting pyroptosis and inhibiting excessive inflammation caused by pyroptosis, and looks forward to provide new insights into the development of promising anti-TB strategies.

## 2. The Role of Pyroptosis in Tuberculosis

**Figure 1:**
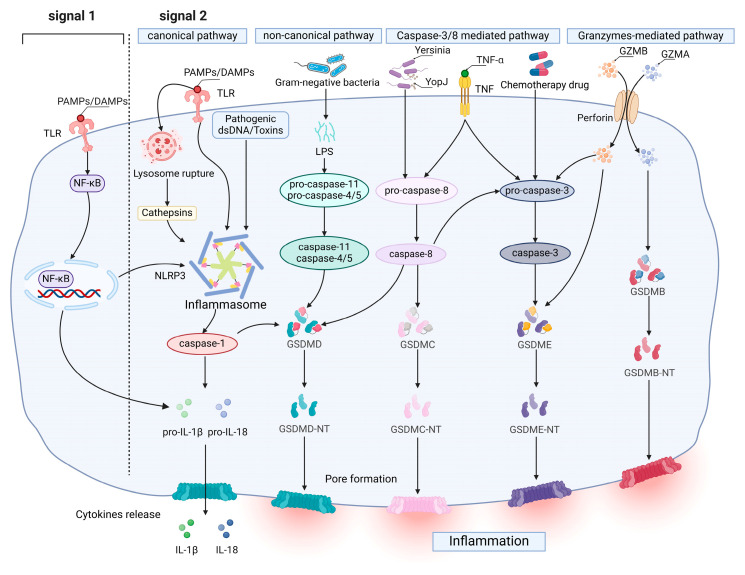
Molecular mechanism of pyroptosis. In the canonical pathway, canonical inflammasome is assembled from intracellular sensor proteins in response to PAMPs and DAMPs. Activated caspase-1 cleaves pro-IL-1β and pro-IL-18, which then leads to the maturation of IL-1β and IL-18 and their subsequent release from GSDMD pores. Activated caspase-1 also cleaves GSDMD, releasing GSDMD-NT to form a non-selective pore at the plasma membrane that releases mature IL-1β and IL-18. In the noncanonical pathway, cytoplasmic LPS activates caspase-4/5 and caspase-11, which in turn cleaves GSDMD and triggers pyroptosis. In the caspase-3/8-mediated pathway, pyroptosis is induced by the Yersinia effector protein YopJ, TNF and chemotherapeutic drugs through the activation of caspase-3/GSDME, caspase-8/GSDMC and caspase-8/GSDMD. In the granzyme-mediated pathway, GZMA and GZMB from cytotoxic lymphocytes can induce pyroptosis by entering cells via perforin and recognizing GSDMB and GSDME, respectively. Created in BioRender. Jia, Y. (2024) BioRender.com/c03x755.

Currently, it is considered that there are four main distinct signaling activation pathways to induce pyroptosis, including the caspase-1-mediated canonical pathway, caspase-4/5/11-mediated noncanonical pathway, apoptotic caspase-mediated pathway and granzyme-mediated pathway (Figure 1). These signaling activation pathways are ultimately directed to cleave gasdermin proteins, which are the final executioners of pyroptosis [22,35,36].

### 2.1. Molecular Mechanism of Pyroptosis

The caspase-1-mediated canonical pathway occurs primarily through the activation of inflammasomes. The activation of the NLRP3 inflammasome requires two independent but accompanying signals: the priming signal (signal 1) can upregulate the expression of proteins associated with inflammasomes (including NLRP3, pro-IL-1β and pro-IL-18) in response to the activation of TLR/TNFR by specific pathogen-associated molecular patterns (PAMPs) or non-pathogen-related damage-associated molecular patterns (DAMPs), inducing the nuclear factor kappa-light-chain-enhancer of activated B cells (NF-κB) pathway [37,38]. The triggering signal (signal 2), a wide range of NLRP3 inflammasome activators, including pathogens, PAMPs/DAMPs, adenosine triphosphate (ATP), etc., triggers NLRP3 inflammasome assembly [39], resulting in self-cleavage and the activation of pro-caspase-1 [25,39]. GSDMD then releases GSDMD-NT for pore formation and cleaves pro-IL-1β and pro-IL-18 to cause an inflammation response and pyroptosis [20,26,40]. The noncanonical inflammasome pathway also forms pores composed of GSDMD-NT, released by cleaving GSDMD to promote pyroptosis [20], but it activate human caspase-4/5 and mouse homologous caspase-11 by direct LPS-binding [41,42,43]. This is required to promote the assembly of the NLRP3 inflammasome by the K+ efflux induced upon cleavage of the GSDMD, thereby causing the maturation and release of IL-1β and IL-18 [44,45,46].

Caspase-3 is activated under the stimulation of TNF or chemotherapeutic drugs; a further study showed that chemotherapy can affect the BAK/BAX-caspase-3-GSDME pathway [47], switching caspase-3-mediated apoptosis to pyroptosis and subsequently directly cleaving GSDME and releasing GSDME-NT for pore formation, which is independent of canonical or noncanonical inflammasomes [48,49,50,51]. And Hu et al. found that BAK or BAX can mediate the pyroptosis pathway individually [47]. In addition to GSDME, apoptotic executioner caspase-3/6/7 can also induce pyroptosis by cleaving GSDMB at the N-terminal domain 88DNVD91 [52]. Furthermore, PD-L1 can convert TNFα-induced apoptosis in cancer cells to pyroptosis through the specific cleavage of GSDMC by caspase-8 [53]. When infected with Yersinia, this leads to the RIPK1- and caspase-8-dependent cleavage of GSDMD, resulting in pyroptosis [54]. Granzymes are a family of serine proteases that are mainly found in cytotoxic T lymphocytes (CTLs) and natural killer (NK) cells. This process shows that granzyme A (GZMA) and granzyme B (GZMB), released by cytotoxic lymphocytes or chimeric antigen receptor-T (CAR-T) cells, could be delivered to the cytoplasm of target cells via perforin [49,55]. GZMA cleaves GSDMB, causing pore formation on the membrane and inducing pyroptosis in target cells that express GSDMB. GZMB released by CAR-T cells can indirectly cleave GSDME by rapidly activating caspase-3 via the caspase3/GSDME-mediated pyroptotic pathway [56].

### 2.2. The Impact of Pyroptosis on Tuberculosis

**Figure 2:**
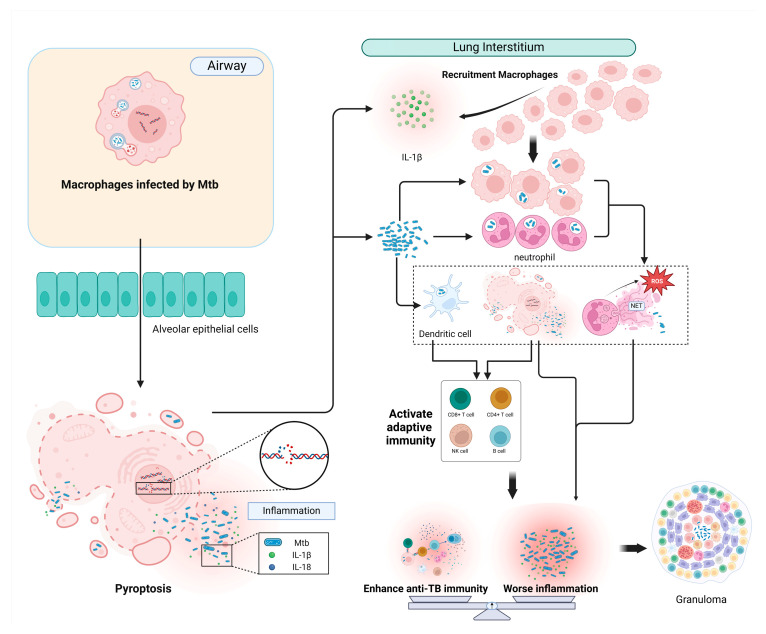
Pulmonary immune responses induced by Mtb-induced pyroptosis. In the airways, Mtb first encounters AMs, and infected AMs migrate to the lung interstitium in a manner determined by the ESX-1 of Mtb and IL-1β of the host. When Mtb enters the interstitium, host cells undergo pyroptosis, releasing IL-1β and IL-18, recruiting more macrophages and triggering an inflammatory response that culminates in cell swelling, DNA breaks and the leakage of cellular membrane content, and the released Mtb infects more macrophage populations and more host cells undergo pyroptosis, which can lead to more severe inflammatory responses and uncontrolled or nonspecific pyroptosis-induced tissue damage and organ dysfunction. Neutrophil populations induce reactive oxygen species (ROS) and neutrophil extracellular traps (NETs) that have little effect on Mtb infection and exacerbate inflammation. Mtb can also be phagocytosed by dendritic cells (DCs), and antigen-transmitting cells link the innate and adaptive immune systems to activate humoral and cellular immunity and ultimately granulomas, limiting the dissemination of Mtb. Created in BioRender. Jia, Y. (2024) BioRender.com/p71v873.

Pyroptosis is typically initiated within cells of the innate immune system, including monocytes, macrophages and dendritic cells. Macrophages represent the primary cellular niche for Mtb during infection, which can identify infection using PRRs (Pattern recognition receptors), which are specific to microbial components such as lipoproteins, glycolipids, virulence proteins, etc. The activation of these receptors triggers a series of pyroptotic events, culminating in the maturation and secretion of cytokines, chemokines and various mediators. These agents subsequently impede infection and expedite the eradication of Mtb. Certainly, as macrophages undergo pyroptosis, their swelling, rupture and subsequent release of cellular content include some surviving Mtb, thereby facilitating bacterial dissemination to adjacent cells (Figure 2).

Type VII secretion system (T7SS), in particular ESX-1, as a key mediator of Mtb pathogenesis, enables certain effector proteins of Mtb to traverse their highly hydrophobic and poorly permeable cell walls via exportation into the cell wall and beyond [57,58]. Concurrent with ESX-1-mediated plasma membrane damage, K+ efflux-mediated NLRP3 inflammasome activation subsequently induces pyroptosis [59]. Macrophages that have received signals from Mtb activate NLRP3/AIM2 inflammasomes, and subsequently, mature caspase-1/11 cleaves GSDMD in a way that allows the release of IL-1β [60,61,62,63]. The results of Cohen, S B. et al. showed that when a subpopulation of Mtb-infected AMs undergoes the ESX-1-mediated activation of inflammasomes and IL-1 release, AMs infected by Mtb but not undergoing activation of inflammasomes are recruited to the lung interstitium by IL-1 released from other airway-resident AMs, and as they accumulate in the lung interstitium, they begin to form granulomas (Figure 2), setting the stage for Mtb to recruit monocyte-derived cells (MCs) and neutrophils, creating the conditions for dissemination [5].

#### 2.2.1. Mtb Effector Involved in Activation of Host Pyroptosis

A range of effector proteins and lipids have been identified as being involved in the regulation of pyroptosis [64]. A secreted Rv1579c protein, encoded by the virulent Mtb H37Rv region of deletion (RD)3, named EST12, interacts with the host protein receptor for activated C kinase 1 (RACK1) to form a complex in macrophages, which recruits the deubiquitinase UCHL5 to stimulate the K48-linked deubiquitination of NLRP3, subsequently contributing to a pyroptosis process of NLRP3 inflammasome-mediated IL-1β release [65]. Additionally, it has been demonstrated that EST12 regulates Myc expression and enhances anti-mycobacterial inflammatory responses via the RACK1-JNK-AP1-Myc immune pathway [66]. The proteins PPE13 and PPE60 both belong to the proline–proline–glutamate (PPE) family; the former promotes the assembly and activation of NLRP3 inflammasomes by directly interacting with the NACHT and leucine-rich repeat sequence (LRR) structural domains of NLRP3 [67] and the latter upregulates pro-inflammatory cytokines produced by macrophages and promotes macrophage pyroptosis through linear ubiquitin chain assembly complex (LUBAC)-mediated NF-κB signaling [68].

**Figure 3:**
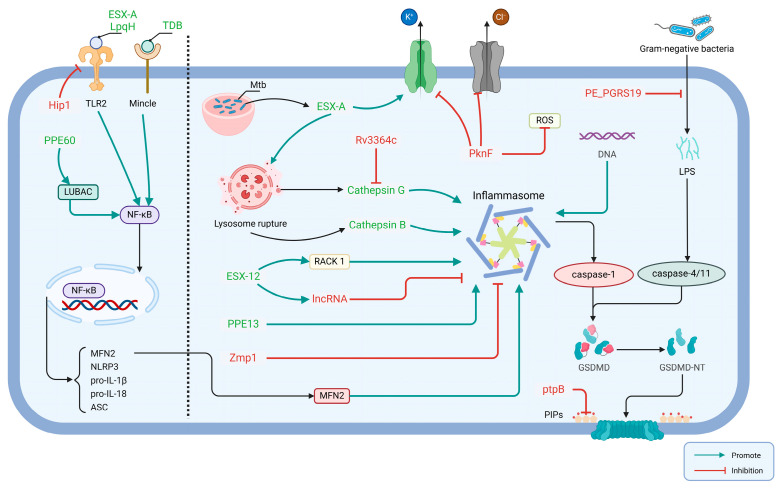
Mycobacterium effectors involved in the regulation of host cell pyroptosis. Several secreted or non-secreted Mtb effectors are known to be involved in the regulation of host cell pyroptosis. Green facilitating arrows indicate Mtb effectors directly or indirectly involved in inflammasome activation, and red inhibitory arrows indicate Mtb effectors involved in the inhibition of the inflammasome. LpqH,19 kDa lipoprotein antigen precursor; ESX-A, 6 kDa early secretory antigenic target; EST12, estimated 12 kDa (Rv1579c); TDM, Trehalose dimycolate; TDB, Trehalose-6,6-dibehenate; Hip1, hydrolase important for pathogenesis 1; PPE13, PPE family protein 13; PPE60, PPE family protein 60; Rv3364c, the serine protease inhibitor; named Zmp1, Zinc metalloprotease-1; MFN2, Mitofusin 2; PknF, Protein kinase F; PE_PGRS19, PE family protein PGRS19; PtpB, protein tyrosine phosphatase B. Created in BioRender. Jia, Y. (2024) BioRender.com/p75x205.

In contrast to PPE13 and PPE60, which induce pyroptosis through the activation of caspase-1, PE_PGRS19 activates the noncanonical pathway to cleave caspase-11, thereby inducing pyroptosis [69]. Furthermore, ESX-A is also partially responsible for releasing mature cathepsin B from lysosomes during Mtb infection, allowing the NLRP3 inflammasome to activate [70,71]. And ESX-A stimulates the delivery of other immunostimulatory Mtb components (e.g., Ag85) into the cell membrane of macrophages, resulting in the enhanced activation of caspase-1 and subsequent secretion of IL-1β [63]. It is noteworthy that the ESX-1 system induces the disruption of the plasma membrane via the K+ efflux-mediated activation of the NLRP3 inflammasome [59]. Mtb lipoprotein LpqH has been demonstrated to activate the NLRP3 inflammasome via a mechanism involving the activation of the TLR-2 receptor, and K+ efflux represents a pivotal trigger of activation [72]. Alginose-6,6′-dibehenate (TDB), a synthetic analogue of alginose-6,6′-dimycolate (TDM), is a mycobacterial cell wall lipid that is recognized by macrophage-inducible C-type lectin (Mincle) receptors on innate immune cells inducing pyroptosis (Figure 3) [73].

#### 2.2.2. Mtb Effector Involved in Inhibition of Host Pyroptosis

Mtb has evolved to become highly adapted to persist within host cells and to regulate host cell death [64], including pyroptosis, through a variety of effectors or pathways. This enables the Mtb to achieve immune escape and to be released from host cells, via a variety of mechanisms, to extend the range of infection.

As for the activation of the AIM2 inflammasome causing pyroptosis, thanks to the fact that AIM2 recognizes any type of dsDNA, it is of interest to note that Mtb does not activate the AIM2 inflammasome when infected with Mouse Bone Marrow-Derived Dendritic Cells (BMDCs) or bone marrow-derived macrophages (BMDMs) [61,74]. The suppression of AIM2 inflammasome activation could represent a pivotal element in the virulence of Mtb, given that mice lacking AIM2 (−/−) have exhibited a heightened susceptibility to infection [61].

Although the regulatory mechanisms and host targets of Zmp1 and PknF to inhibit the inflammasome–pyroptosis pathway remain undefined, the Mtb serine threonine kinase, PknF, belongs to the 11-member family of Mtb serum/threonine protein kinases [75]. Rastogi S et al. verified that a lack of PknF in the PknF-Rv1747-PIMs-NLRP3 pathway fails to limit NLRP3 inflammasome activation [76]. The PknF-mediated inhibition of the NLRP3 inflammasome was also associated with K+ efflux, Cl- efflux and ROS generation [76]. The latest research conducted by Rastogi et al. has demonstrated that Mtb employs the pknF mechanism to evade NLRP3 inflammasome-mediated activation of the caspase-1 and RIPK3/caspase-8 pathways in mouse dendritic cells [77]. Mtb serine hydrolase Hip1 inhibits the activation of the NLRP3 inflammasome by suppressing TLR2-dependent cell signaling in BMDMs [78]. In addition, the Rv3364c protein from Mtb binds to the membrane-associated host serine protease cathepsin G, which results in a downstream of caspase-1 activity and pyroptosis.

Zinc metalloprotease-1, named Zmp1, from Mtb has been shown to inhibit the NLRP3 inflammasome-dependent processing of IL-1β [79]. Nevertheless, it has been identified that the production of ZMP1-deficient Mtb mutant strains has no effect on pyroptosis, caspase-1 activation and IL-1β release [60]. This could be since most of the studies by Master et al. were undertaken using the BCG strain, which in contrast to Mtb lacks a functional ESX-1 secretion system, while the latter may have been used to assess differences in the cell types of the ZMP1-deficient strains, and it is possible that these reasons led to different conclusions in the two studies. Yao et al. identified a key long-chain non-coding RNA (lncRNA), negatively regulated by EST12, which inhibits macrophage pyroptosis and promotes Mtb survival [80]. In a recent study by Q. Chai et al., it was shown that protein tyrosine phosphatase B (PtpB), a eukaryotic-like protein secreted by Mtb, possesses both protein phosphatase and lipid phosphatase activities [81]. Moreover, the study revealed that PtpB interacts with host ubiquitin, enhancing its phosphatidylinositol phosphatase activity. This interaction leads to the targeting and dephosphorylation of host plasma membrane phosphatidylinositol, consequently inhibiting membrane localization and the pyroptosis of GSDMD-N (Figure 3) [17].

It is still controversial whether pyroptosis is more likely to fight infection or contribute to the Mtb process in TB. Host cells infected with Mtb utilize pyroptosis as a mode of cell death to secrete cytokines that recruit immune cells to reach the lesion, cause local inflammation or even “die with Mtb”, thus promoting the process of killing Mtb and controlling the infection. However, pyroptosis frequently results in the release of cellular contents, which inevitably releases the surviving Mtb and expands the scope of infection. Meanwhile, non-selective or uncontrolled pyroptosis can exacerbate the inflammatory response, leading to severe tissue damage and organ dysfunction, which may worsen the outcome of TB infection (Figure 2).

In summary, host cells engaged in pyroptosis can contribute to the control of Mtb infection through cell lysis and the secretion of inflammatory factors, recruitment of immune cells and promotion of granuloma formation, but at the expense of immune cells and with the potential to induce a severe inflammatory response. For Mtb, it is undoubtedly a process of broadening the scope of infection, and the Mtb released when pyroptosis occurs further infects more immune cells. Consequently, pyroptosis in TB can be regarded as a “double-edged sword”, and more attention should be paid into the studies of pyroptosis in Mtb infection and TB progression. Although it is an important component of the host defense mechanism against TB, its dysregulation or over-activation can exacerbate tissue damage and contribute to the severity of TB disease. It is crucial to comprehend the balance between the beneficial and detrimental effects of pyroptosis in TB immunity if therapeutic strategies are to be developed that can harness its protective effects while mitigating its harmful consequences.

## 3. Potential of Pyroptosis as Anti-TB Target

In infectious diseases, cell pyroptosis has emerged as a key host antimicrobial response mechanism that is regulated bidirectionally by both the host and the pathogen to determine the outcome of infection [82]. A growing body of research suggests that modulation of the pyroptosis pathway may be a promising therapeutic approach against infectious diseases [83]. It has been demonstrated that certain bacteria can exert direct killing effects via the pyroptosis pathway [84,85,86,87]. For example, Crowley et al. showed that pyroptosis is essential for the control of Salmonella typhimurium infection in intestinal epithelial cells, and that it prevents Salmonella from expanding its infectious ecological niche while further strengthening host defenses [88]. However, some specific pathogens, such as Mtb, have evolved a complex cell-wall envelope to evade pyroptosis-induced killing effects and worsen pyroptosis-mediated inflammatory responses, thus facilitating their infection [89]. And as pyroptosis may cause uncontrolled inflammations in Mtb-infected tissue sites, pyroptosis has been identified as a “double-edged sword” in the context of Mtb infection.

Since it is difficult for pyroptosis to exert a direct killing effect on Mtb and the effect of pyroptosis alone on Mtb is limited, it is conceivable that pyroptosis could be used to interact with and cross-regulate other types of cell death programs to create a complex network of cell death involved in the onset and development of TB. Examples include autophagy, apoptosis, ferroptosis, cuprotosis, PANoptosis, which has attracted attention in recent years, and so on.

As the current World Health Organization (WHO)-recommended TB drug combination therapy is associated with a long course of treatment, multiple drug administrations, and multiple and extensive drug resistance, there is a need to explore newer therapeutic options that can overcome the drawbacks of the regimens, and novel nanocarriers based on nanotechnology provide new ideas for the treatment of TB. Nanocarrier-mediated drug delivery systems are submicron-sized, versatile vehicles in which the drug can be encapsulated [90,91,92,93,94,95]. The biological basis of the versatility of nanomaterials in targeting, controlled drug release, self-administration, efficient transport and other multifunctionalities, as well as multiple regulatory mechanisms [96,97,98,99,100], has enabled the development of numerous pyroptosis-carrying drug delivery carriers or pyroptosis-regulated functional agents that have the potential to regulate pyroptosis [101,102,103,104,105]. Based on the results of combining existing anti-TB drugs, it is possible to design novel anti-TB therapies combining host cell pyroptosis regulations to achieve enhanced anti-TB therapy. In the development of novel TB treatments targeting pyroptosis, more attention can be paid to the use of re-purposing old drugs and natural products, which possess anti-inflammatory effects that have the potential to inhibit excessive inflammatory responses caused by uncontrolled pyroptosis, so that they favor the killing process of Mtb [106,107]. Host-directed therapy (HDT) based on the enhancement of protective immunity or the attenuation of immune-regulatory responses in the advanced stages combined with known first-line drugs for treating TB plays an auxiliary role in TB treatment [108], which can shorten the duration of the course of treatment and prevent the recurrence of TB [109]. For example, Fu et al. demonstrated that andrographolide, a bioactive compound derived from Andrographis paniculata, inhibits excessive inflammatory responses associated with pyroptosis by preventing macrophage pyroptosis in Mtb infections via the microRNA-155/Nrf2 axis [110], and Ning et al. found that Baicalein inhibits the classical pathway of pyroptosis in macrophages infected by Mtb by inducing autophagy [111]. The use of medicinal plants to modulate inflammasomes may facilitate more effective management of Mtb infections through HDT, which assists in reducing the adverse effects of pyroptosis on surrounding tissues; this hypothesis is also outlined in the review by Cebani and Mvubu showing the potential to enhance the anti-TB efficacy of current chemotherapy [112]. And the exploitation of functional nanomaterials to eradicate Mtb and reduce pathological damage and exhibit anti-inflammatory activities in the lungs also offers superior therapeutic potential compared to first-line antibiotic combinations [113]. Vitamin D3 is a fat-soluble vitamin, and vitamin D3 inhibits pyroptosis by inhibiting the AMPK/NLRP3 inflammasome pathway [114], which is also a good HDT strategy. IL-18 promotes the production of interferon IFN-γ by Th1 cells, NK cells and cytotoxic T cells, which is a key factor in the anti-tuberculosis immune process and promotes the development of Th2 cells and local inflammatory responses [115]. In addition, it would be a promising therapeutic target if the process of IL-18-induced IFN-γ production could be enhanced at the time of pyroptosis, while at the same time lessening the inflammatory response by inhibiting excessive pyroptosis.

As mentioned above, many Mtb effectors inhibit the pyroptosis pathway, and if the inhibition of pyroptosis during bacterial infection could be removed, it might enhance the early protective immune responses of the host. However, another important thing is how to control the inflammatory responses induced by pyroptosis, which may need combined treatments to avoid uncontrolled inflammations that worsen TB progression. Furthermore, improving downstream immune processes, starting with the link between focal death and adaptive immunity, would also be a new target.

## 4. Conclusions

In summary, TB remains a major and important disease impairing public health around the world. Pyroptosis, a mode of programmed cell death that causes cells to swell until the cell membrane ruptures, releasing cellular content and activating a strong inflammatory response, plays a role in innate immune defenses against Mtb infection and TB disease progression. Although significant progress has been made in the discovery of the mechanisms of host cell pyroptosis upon Mtb infection, the current knowledge in the field is not comprehensive enough to identify the relevant roles and mechanisms of pyroptosis in TB. In the future, refining as much as possible the mechanisms by which Mtb regulates pyroptosis, especially the immune escape mechanisms of Mtb by manipulating host cell pyroptosis, will be beneficial in providing new ideas for the discovery of new targets in innate immunity against Mtb infection and laying an important foundation for the design of novel TB vaccines and therapeutic regimens. Given that pyroptosis is a “double-edged sword” in TB, we believe that the development of therapeutic strategies for TB using pyroptosis must consider the balance between promoting pyroptosis-mediated host defenses and preventing uncontrolled or nonspecific pyroptosis-induced tissue damage and organ dysfunction that may exacerbate the severity of the disease. Finding a balance between these two factors is a primary focus that cannot be ignored when exploiting the pyroptosis pathway to develop anti-TB treatments. If this balance can be addressed well, we believe that novel therapeutic strategies can be developed by targeting pyroptosis pathways for effective anti-TB treatments.
